# Facilitators and barriers to voluntary medical male circumcision as an HIV prevention strategy in Kavango East, Namibia

**DOI:** 10.4102/safp.v65i1.5684

**Published:** 2023-05-10

**Authors:** Daniel O. Ashipala, Trymore B. Nhokwara, Medusalem H. Joel

**Affiliations:** 1 Department of General Nursing Sciences, School of Nursing and Public Health, Faculty of Health Sciences and Veterinary Medicine, University of Namibia, Rundu, Namibia

**Keywords:** voluntary, medical male circumcision, facilitators, barriers, prevention, strategy

## Abstract

**Background:**

Voluntary medical male circumcision (VMMC) is a strategy used to try to limit new human immunodeficiency virus (HIV) infections, as it has the potential to reduce HIV and/or AIDS transmission from women to men by up to 60%. However, in spite of efforts by the Ministry of Health and Social Services, only a few men in Namibia have been circumcised. The objective of this study was to explore and describe the facilitators of, and barriers to, medical male circumcision for HIV prevention in Kavango East, Namibia.

**Methods:**

A qualitative, explorative, descriptive and contextual design was employed. The accessible population in this study comprised 18 health professionals who were selected for the study using a purposive sampling technique.

**Results:**

Participants reported numerous barriers to VMMC in Namibia. Barriers to VMMC included ‘myths’ and misconceptions attached to VMMC, age limitations, fear of pain and stigma associated with HIV, small VMMC team and long distances from health facilities. Facilitators to VMMC included family support, having experienced genital sores and genital warts or phimosis and paraphimosis.

**Conclusion:**

The study revealed that a number of barriers must be overcome before VMMC before the desired number of men take advantage of VMMC. Multiple factors act as constraints to VMMC, including fear, myths and misconceptions, small VMMC teams and the long distance between clients’ homes and VMMC services.

**Contribution:**

The study’s findings can be used to develop targeted interventions and strategies that can be used by VMMC providers to address the identified barriers.

## Introduction

Human immunodeficiency virus (HIV) remains one of the most significant public health challenges globally, especially in sub-Saharan Africa. For that reason, the World Health Organization (WHO) and Joint United Nations Program on HIV and AIDS (UNAIDS) have recommended the implementation of voluntary medical male circumcision (VMMC) in countries such as Namibia with a high prevalence of HIV.^1^ Voluntary medical male circumcision, the surgical removal of the foreskin covering the head of the penis, is one of the most ancient and common surgical procedures worldwide.^2^ As indicated in a study conducted by George et al.,^3^ VMMC can reduce HIV transmission from women to men by up to 60%, and also plays a key role in reducing the likelihood of cervical cancer among female partners. In the UN’s 2016 Political Declaration on HIV and AIDS, a new VMMC target was set that aimed to ‘reach 25 million young men in priority countries by 2020’.^3^ A WHO/UNAIDS VMMC strategic framework subsequently set a goal to reach 90% of 10 to 29-year-olds in priority countries by 2021.^4^ To achieve this, 5 million males in the listed countries would have needed to volunteer to undergo circumcision every year.^4^ Research has revealed that approximately 30% of the world’s male population aged 15 years and above are circumcised. Of these, approximately two-thirds (69%) are Muslims (living in Asia, the Middle East and North Africa), 0.8% are Jewish and 13% are non-Muslim/non-Jewish men living in the United States.^5^

With a population of only 2.5 million, Namibia has seen some success in lessening the prevalence of HIV, as well as reducing the mortality rate from AIDS,^6^ yet Namibia is still ranked in the top 10 countries globally with the highest rates of HIV.^7^ As of 2015, an estimated 178 000 Namibians were believed to be HIV positive.^8^ Although antiretroviral therapies are available and the government has instituted various HIV prevention and control measures, the level of new HIV infections remains high. Possible causes of Namibia’s high level of HIV infections include multiple and concurrent sexual partnerships; intergenerational sex; alcohol use; low condom use; transactional sex; population mobility; early age at sexual initiation; and, central to this analysis, low rates of male circumcision.^9^

The VMMC programme was initiated in 2009 in Namibia, as observed by Nairenge,^9^ in order to be:

[*U*]sed in conjunction with other HIV prevention methods such as HIV testing and counselling, correct and consistent [*sic*] use of female and male condoms, [*and the*] provision of antiretroviral treatment for people living with HIV. (p. 3)

A total of 14 African countries are utilising the VMMC programme, with Namibia signing on in the light of its high rate of HIV infections and low rate of circumcision. According to the Namibia Country Operational Plan (COP),^10^ data suggests that uptake of VMMC below age 15–49 years is lower than expected, given the age distribution of the eligible population. Modelled national coverage for VMMC among priority age groups of 15–29 years old is 47.7% (Decision Makers Program Planning Tool, DMPPT-2 2019), which is less than the recommended 80% target to achieve a population level impact in epidemic control. Since then the (Ministry of Health and Social Services [MoHSS]) has embarked on intensifying VMMC demand creation campaign. This was part of larger campaign efforts, from the part of President’s Emergency Plan for AIDS Relief (PEPFAR) Namibia, in collaboration with MoHSS, to promote the benefits of VMMC and thereby increase uptake of VMMC among young people aged 15–29 years.

The reasons why men in the 14 countries chose to reject a circumcision were discussed in a 2013 scoping report,^11^ with the most frequent reasons listed as fear of pain during and after the surgery; costs (both explicit and implicit); concerns about adverse events or complications relating to surgery; threats to masculinity, including loss of penile sensitivity or penis size; concerns about sexual performance; sexual inactivity; and religious concerns. Concerns about undergoing an HIV test were found in an additional study to be a major cause of concern,^12^ with a third study noting social barriers involving ‘culture or tradition and ethnic identity’.^11^ Conversely, it has been observed that individual level facilitators of VMMC include: hygiene; protection from sexually transmitted infections (STIs); sexual performance and satisfaction; ease of condom use and acceptability by other ethnic groups. Peer pressure and preferences of female intimate sexual partners are the primary social level facilitators.^11^

Government clinics are subsidised to offer VMMC services in regions not supported by the President’s Emergency Plan for AIDS Relief and Global Fund to Fight AIDS, Tuberculosis and Malaria (PEPFAR/GFATM). This financing emanates from the Health Facility budget.^13^ In addition, AIDS-free supports private doctors who offer VMMC to private clients. An AIDS-free generation is a time when, first of all, virtually no child anywhere will be born with the virus. Secondly, as children and teenagers become adults, they will be at significantly lower risk of ever becoming infected than they would be today, no matter where they are living. And thirdly, if someone does acquire HIV, they will have access to treatment that helps prevent them from developing AIDS and infecting others with the virus.^14^

In 2008, the International Training and Education Centre (I-TECH) began supporting the MoHSS in the expansion and provision of VMMC as an HIV prevention alternative. This support entails the national training of healthcare workers and the delivery of services in the Oshana and Zambezi regions. Subsequently, in early 2016, I-TECH started supporting VMMC demand creation through a network of community-based mobilisers and recruiters, who engaged local and regional stakeholders in an attempt to boost the number of men who volunteer to undergo medical circumcision.^12^ To date, I-TECH has trained nurses (registered and enrolled), physicians and community counsellors to make certain that sufficient ‘skills and experience are in place to deliver safe, high-quality, male circumcision services’.^15^ Despite all these efforts, however, in Kavango East Region, out of 62 102 men, only 2 418 men were circumcised in the year 2022, which shows a low VMMC uptake in this region. This modest uptake of VMMC implies that some contributing factors may be overlooked in the provision of VMMC services to consumers. In addition, the low uptake of VMMC, as a measure for preventing HIV transmission, implies a necessity for increasing the utilisation of VMMC services to the maximum. It is thus important to strengthen the implementation of this HIV prevention strategy.

Although several studies have been conducted on VMMC, with some focusing on assessing the impact of age prioritisation on circumcision^6^ and others on assessing the knowledge, practices, attitudes and responsiveness of males towards VMMC,^9,14^ none of these has focused on exploring specifically the facilitators of, and barriers to, VMMC in particular in Namibia. Namibia is a culturally diverse nation; therefore, exploring the barriers and facilitators to VMMC can provide useful information regarding the low uptake of VMMC.

### Research aim

The aim of this study was to explore and describe the facilitators of, and barriers to, medical male circumcision for HIV prevention in Kavango East region, Namibia, with a view to describing aspects that affect the uptake of VMMC by men in the region.

## Research methods and design

The study used a qualitative, exploratory and descriptive design to explore the meaning of the experiences of the facilitators of, and barriers to, effective VMMC uptake. According to Hennink, Hutter and Bailey,^16^ ‘qualitative studies focus on the meaning and understanding of experiences as lived by participants’, while Flick^17^ described an explorative research design as the ‘identification of problems within a certain practice and justification of that practice’. As per Cardano,^18^ the process of inquiry for this study ‘involved the researchers conducting the study in a natural setting and developing a complex holistic picture, as well as analysing and reporting the detailed views of the participants’. This study thus focused on the meaning and understanding of the participants’ experiences as they experienced them. The aim of qualitative research is to gain an understanding of a certain phenomenon, rather than to explain or predict it;^19^ therefore, the researchers conducted semi-structured individual interviews in order for them to make sense of the participants’ experiences. Preventing HIV transmission through VMMC is a complex and multifaceted phenomenon; therefore, a qualitative design was selected to gain a detailed, in-depth understanding of the facilitators and barriers.

### Setting

This study was conducted at Kavango East Hospital, a Rundu Intermediate Hospital located in the north east, Namibia. Rundu is a rural area with approximately 115 447 inhabitants; it is the second largest city in Namibia after the capital, Windhoek. The town is the main centre of the Kavango East region. With the country’s highest poverty rate (53.2%), 64% of its population is materially deprived, while 29.6% are unemployed.^20^ The above-mentioned facts, combined with the low literacy levels of the Kavango East population, might have contributed to an increase in unprotected sexual relations, hence its high HIV prevalence rate of 16.9%. Yet out of 62 102 men, only 2 418 were circumcised in the year 2022. For this reason, Rundu Intermediate Hospital was made the headquarters of VMMC in the region. This is also where regional VMMC outreach programmes are being organised, whereby professionals are grouped together, before being equipped with the necessary resources and sent out to different places within the region for circumcision procedures. This programme promotes the accessibility of VMMC services for all men within the region, regardless of their social status or geographical location, so as to counter the high HIV rate.

Rundu Intermediate Hospital, with a bed capacity of approximately 300, is the largest hospital in Kavango East region, which caters for referrals from the Kavango East regions. The health facility also serves a large number of Angolan patients who live along the Kavango River. The VMMC department comprises surgeons, registered nurses, enrolled nurses, health assistants and a quality assurance officer.

### Population and sample

The target population was healthcare workers working in the VMMC programme. A purposive sample of 18 healthcare workers – 12 males and six females – who worked at the VMMC centre was drawn to participate in the study. A purposive sampling technique was chosen for this study because it produces substantial results in real-time while conducting human experiments, as these people already had some specific knowledge about the research topic. In addition, it allows one to target niche demographics to obtain specific data for the research and lowers the margin of error in the data because the data sources are a close fit with the research context. Inclusion criteria included trained health professionals registered with (Health Profession Council of Namibia) HPCNA, who had at least 1-year work experience in a VMMC department, who were working within Kavango East region, Namibia and were willing to participate by signing an informed consent. Data saturation was reached when the 18th interview yielded no new information.

### Data collection

In this study, data were collected during August 2020 and September 2020 after approval had been granted by the School of Nursing Ethics Review Committee and the Ministry of Health and Social Services Institutional Review Board. The semi-structured interviews were conducted by the same interviewer at the Kavango East region department in the Kavango East region in accordance with an interview guide, which was developed based on the research question and the literature review. The interview guide comprised the following sections: Section A and Section B. Section A requested socio-demographic information from the participants in order to contextualise the research findings in terms of their background. Section B consisted of three questions:

- What are the facilitators to VMMC as an HIV prevention strategy in Kavango East region, Namibia?
- What are the barriers to VMMC as an HIV prevention strategy in the Kavango East region, Namibia?
- What can be done to improve VMMC uptake in Kavango East region, Namibia?

Data saturation was reached at 18 participants, thus no further interviews were conducted. Each interview lasted between 30 min and 34 min.

### Data analysis

Data from the audio recordings were transcribed verbatim before being analysed using qualitative thematic analysis.^21^ The transcripts and narratives were thematically analysed following Braun’s six-step method of data analysis, which included: (1) organising and preparing data; (2) developing a sense of all the data; (3) coding data following Tesch’s nine steps; (4) identifying and describing themes; (5) representing the findings; and (6) interpreting the data.^21^ The researchers and the independent coder then held a consensus discussion and agreed on the main themes and sub-themes that emerged. The analysed data are presented using the main research questions and objectives as the key themes.

### Measures to ensure trustworthiness

Trustworthiness in this study was ensured through the application of the credibility, transferability, dependability and conformability criteria proposed by Lincoln and Guba.^22^ During the 2 months spent by the researcher with the participants until data saturation was reached, an in-depth understanding of phenomenon was gained, thus ensuring credibility. Before the actual data collection, the interview guide was tested on three participants who were also part of the study; however, no changes were made to the interview guide. The researcher ensured transferability through a thick description of the data collection process, as well as using verbatim transcriptions of the voice recordings and field notes. For dependability, the research method was reported in detail to indicate that proper research practices were followed and that the study was replicable.^23^ Dependability was also facilitated by means of literature control and prolonged engagement, as well as by a step-by-step ‘audit trial’.^24^ To guarantee confirmability, the interviews were recorded, ensuring that study’s findings were the results of the respondents’ experiences and ideas as far as possible. An independent coder was used to confirm the data collected via the interview guide, which the researcher in turn confirmed to ensure that the data were accurate and in line with the information provided by the participants.

### Ethical considerations

Before conducting the study, institutional approval was sought from the Ministry of Health and Social Services (MoHSS) (Reference number 17/3/3 TBN). Ethical approval was also obtained from the Health Research Ethics Committee of the University of Namibia (UNAM) (Reference no. SoNREC 15/2020). In addition, approval was obtained from the permanent secretary, Ministry of Health and Social Services in Namibia, and the head of Rundu Intermediate Hospital, VMMC department. Written informed consent was obtained from all participants. The researchers were guided throughout the study by the following ethical principles: ‘beneficence, non-malfeasance, confidentiality, privacy, anonymity, justice, informed consent and autonomy’. The interviewees’ data were only made available to the researchers. In addition, the respondents were assured of ‘privacy, anonymity, confidentiality and their right to terminate their participation in the study at any point without having to explain themselves or incur penalties for doing so’.^25^

## Results

### Participants’ demographic data

A total of 18 participants were interviewed, all of whom were health professionals working in the VMMC programme. Of the 18 respondents, 12 (67%) were men and 6 (33%) were women. All fell within an age range of 26–40 years. The characteristics of the study participants are given in Table 1.

**TABLE 1:** Characteristics of the study participants.

Characteristics	Total
**Age**	
26–30	6
31–35	6
36–40	6
**Gender**	
Female	6
Male	12
**Profession**	
Quality assurance officer for VMMC	1
Registered nurse	6
Health assistant	6
Surgeon	5

VMMC, voluntary medical male circumcision.

### Presentation and discussion of findings

The three themes that emerged from the data analysis as indicated in Table 2 were: (1) enablers for the uptake of VMMC by males; (2) challenge of barriers to motivate males to VMMC uptake and (3) recommendations to increase the uptake of VMMC. These themes have several sub-themes.

**TABLE 2:** Summary of findings.

Themes	Sub-themes
Enablers for the uptake of VMMC by males	Positive effects
	Health education
	Family support
	Physical penile problems
	Phimosis and paraphimosis
Challenge of barriers to motivate males to VMMC uptake	Myths and misconceptions
	Age limitations
	Fear of pain and stigma associated with HIV
	Small VMMC team
	Long distances from health facilities
Recommendations to increase the uptake of VMMC	Employing more health professionals in the VMMC programme
	Strengthening the current programmes to motivate males to seek medical attention
	Massive education to clear up myths and misconceptions
	Circumcision of boys at a young age before they are sexually active
	Opening of more VMMC centres in the region

VMMC, voluntary medical male circumcision; HIV, human immunodeficiency virus.

#### Theme 1: Enablers for the uptake of voluntary medical male circumcision by males

This theme is a description of the participants’ experiences regarding the facilitators of VMMC. Five sub-themes that emerged from this theme were as follows: positive effects, health education, family support, sores and genital warts, as well as phimosis and paraphimosis.

**Sub-theme 1: Positive effects:** The participants revealed that most clients came for VMMC because of the benefits it offers:

‘What attracts them is the fact that VMMC protects men from contracting HIV by 60%’. (P5, 31 years old, Surgeon)

‘Most of them tells us that they want to protect their partners from getting cervical cancer that is why they choose to be circumcised’. (P7, 34 years old, Registered Nurse)

It emerged from this study that there are some factors that facilitate VMMC. The respondents indicated that positive factors associated with VMMC include a reduction in HIV transmission by 60%, preventing females from getting cervical cancer, and improving penis hygiene. A study conducted by Morris and Hankins^26^ exploring the effects of male circumcision on the risk of STI and cervical cancer in women similarly revealed that circumcision reduces HIV transmission by 60%, which is the most common reason for men choosing it. Furthermore, a study by George et al.^3^ found that many men opt for circumcision because it protects their female partners from cervical cancer and improves penis hygiene.

**Sub-theme 2: Health education:** Some of the participants highlighted that the health education that they give to males via various media platforms such as Facebook, Twitter and radio stations, as well as the health talks that they do before the procedure when they highlight some of the benefits and possible side effects of VMMC, attracted more males:

‘Some will tell you that I got information about VMMC on Facebook; that’s why I came’. (P9, 37 years old, Registered Nurse)

‘We normally do health talk before the procedure and that’s when most men make up their minds and decide to get circumcised’. (P18, 35 years old, Surgeon)

This is in line with a study conducted by Avert,^27^ which revealed that health education regarding VMMC plays a significant role in attracting people to the procedure. In addition, a study conducted by Maibvise and Mavundla^28^ in Swaziland found that transforming men’s mindsets about male circumcision promotes the uptake of VMMC.

**Sub-theme 3: Family support:** The participants indicated that family support also played a role as a facilitator of VMMC. According to the VMMC service providers, most clients acknowledged that they were encouraged by their friends, siblings or partners to be circumcised:

‘Some clients would say their partners encouraged them to get circumcised because they prefer a circumcised man’. (P1, 26 years old, Registered Nurse)

‘Most men come for circumcision because they were recommended by their siblings and friends who were already circumcised’. (P10, 27 years old, Health Assistant)

Similar findings were found in the study conducted by Maibvise and Mavundla,^29^ which indicated that some men undergo male circumcision primarily for psychosocial motives such as giving in to pressure from sexual partners and peers. A study conducted in Lesotho by Skolnik et al.^5^ also found that most men chose to be circumcised because of the support they got from their family and friends.

**Sub-theme 4: Physical penile problems:** The participants revealed that some clients come for circumcision because they want to get rid of sores and genital warts:

‘Some clients will tell you that they are only getting circumcised because they want to get rid of genital [*problems*]’. (P4, 38 years old, Quality Assurance Officer for VMMC)

‘Some males would say that if it was not for sores and genital warts they wouldn’t have been circumcised’. (P15, 33 years old, Health Assistant)

Similarly, a study conducted by Ashengo et al.^30^ on VMMC in Tanzania and Zimbabwe found that some men were motivated to undergo male circumcision for medical reasons such as congenital malformations, for example, when the foreskin is too big or is fragile and prone to tearing, leading to pain.

**Sub-theme 5: Phimosis and paraphimosis:** One participant indicated that some clients come for circumcision as a treatment for phimosis and paraphimosis:

‘Conditions like phimosis and paraphimosis require circumcision and there is no other way on that’. (P16, 31 years old, Surgeon)

Similar findings emerged from Maibvise and Mavundla’s^31^ study on medical reasons for performing adult male circumcision in Swaziland, which indicated that some men get circumcised not because they want to do so, but as a treatment for phimosis and paraphimosis.

#### Theme 2: Challenge of barriers to motivate males to voluntary medical male circumcision uptake

This theme is a description of the participants’ responses when they were asked to share their experiences about the barriers to males accessing VMMC. Based on the data collected, the following sub-themes were generated: myths and misconceptions; age limitations; personal factors; small VMMC team and long distances from health facilities.

**Sub-theme 1: Myths and misconceptions:** According to the participants, the biggest barrier to VMMC is mistaken beliefs or wrong ideas about the procedure. Some men believe that if they get circumcised, some misfortune will befall them or their sexual life will change for the worse:

‘Some clients revealed that they cannot get circumcised because they believe that after circumcision the sexual pleasure will be diminished’. (P6, 40 years old, Surgeon)

‘One client told me that he does not want to get circumcised because if the foreskin is removed the penis will develop some cracks’. (P16, 31 years old, Surgeon)

Other myths and misconceptions include that some men believe that if they are circumcised their firstborn will die, while others said that they should only be circumcised in winter when the wound would heal more rapidly. This finding is in line with a study conducted in Uganda by Kibira et al.,^15^ who found that most men refuse to be circumcised because of the myths that they hear from others or their own misconceptions concerning circumcision.

**Sub-theme 2: Age limitations:** One participant (a surgeon) raised a concern that a limitation is set as there is a minimum age at which a male can be circumcised. The surgeon added that they are only allowed to circumcise males from 15 years old and they are now getting fewer clients than before:

‘Due to the new developments, we can only circumcise males from 15 years of age and because of that we are now getting fewer clients’. (P5, 31 years old, Surgeon)

Participants in this study reported that they only circumcise males of 15 years and above. These findings are supported by those that were obtained in a study conducted in Zimbabwe on early infant male circumcision by Mavhu et al.,^32^ which indicated that most people do not agree with the idea of circumcising infants as they believe only those who are old enough to take care of themselves should be circumcised. This is in contrast to some reports of the progress in male circumcision has been reported where eight countries experienced an increase in the proportion of clients in the < 15 years age ranges from 2016 to 2017. This shift was largest in Rwanda (5% – 28%), Botswana (45% – 66%) and Namibia (12% – 34%), all relatively small programmes Davis et al.^33^

**Sub-theme 3: Fear of pain and stigma associated with HIV:** The participants felt that personal factors also play a role in hindering VMMC:

‘Some men are afraid of the stigma associated with HIV so they choose to stay without knowing their status’. (P6, 40 years old, Surgeon)

‘Generally, males have lower health-seeking behaviour than women and are less likely to access HIV prevention’. (P14, 30 years old, Registered Nurse)

Other personal factors that hinder VMMC include a fear of pain associated with injections, the procedure and the wound healing process, as well as fear of HIV testing. In addition, some males are not prepared to abstain from sex for 6 weeks after the circumcision procedure. Furthermore, some men do not believe that they are at risk of getting HIV, and generally males have lower health-seeking behaviour than women and are less likely to access HIV prevention. These findings are supported by those of Nairenge,^9^ who revealed that some men do not want to be circumcised because they are afraid of surgery as well as the pain experienced during the wound healing period. Another study conducted in Malawi by Masese et al.^34^ stated that some males do not believe that they are at risk of contracting HIV, which is why they do not bother to go for VMMC.

**Sub-theme 4: Small voluntary medical male circumcision team:** The participants expressed the concern that their team is very small and it is difficult for them to reach each and every person:

‘At the moment the whole of region circumcision is only done in Kavango East so we might take about six months before we go to other places’. (P1, 26 years old, Registered Nurse)

‘We are very few and it is very difficult to cater for the whole Kavango East Regions’. (P14, 30 years old, Registered Nurse)

Participants in this study reported that the VMMC is small to cover the region. Curran et al.^35^ reported that one of addressing human resources in VMMC can be through the maximisation through; identifying task shifting, task sharing, temporary redeployment of public sector staff during the times of VMMC campaigns, extension of the health workforce through recruitment of unemployed, recently retired and newly graduates. In addition, Mahler et al.^36^ also proposed the following efforts in matching the supply and demand for VMMC, which include focusing on community-driven demand creation, as well as through ensuring efficient site-level client flow; by adding needed counsellors and creating more space for VMMC, proper scheduling, detailed logistics, and the adoption of surgical efficiencies.

**Sub-theme 5: Long distances from health facilities:** The participants revealed that some males fail to obtain VMMC services even if they want to because they live a long distance from where the service is being provided:

‘Some people would really want to get circumcised but they stay very far so they will end up not coming’. (P1, 26 years old, Registered Nurse)

‘Some males stay more than 200 km away so they cannot afford to come for circumcision’. (P4, 38 years old, Quality Assurance Officer for VMMC)

The findings of this study are supported by research undertaken by Nairenge^9^ in the Zambezi region, Namibia, which confirmed that many men are discouraged from seeking medical male circumcision because they stay too far away from the facilities where it is provided and they cannot afford transport to go there. Similarly, the findings of a study by Nyoni^37^ revealed the high cost of travelling to access circumcision services, that is, having fewer VMMC service providers’ acts as a barrier to VMMC.

#### Theme 3: Recommendations to increase the uptake of voluntary medical male circumcision

This theme is a description of what the participants mentioned when they were asked to suggest what could be done to increase VMMC uptake. The sub-themes in this theme are as follows: employing more health professionals in the VMMC programme; launching programmes to encourage males to seek medical attention; more education to clear up myths and misconceptions; circumcision for boys at a young age before they are sexually active and opening more VMMC centres in the region.

**Sub-theme 1: Employing more health professionals in the voluntary medical male circumcision programme:** Some participants suggested that more health professionals should be employed in the VMMC programme so that there are enough teams to reach everyone who requires it:

‘There is need for more surgeons’. (P5, 31 years old, Surgeon)

‘More health assistants and surgeons should be employed’. (P13, 26 years old, Health Assistant)

It emerged from this study that increasing the number of VMMC service providers, including nurses, surgeons and health assistants, could be helpful in ensuring that the VMMC is delivered to all parts of the country, thereby increasing its uptake. Similar findings emerged from a study conducted by Mavhu et al.^38^ on ‘innovative demand creation strategies to increase VMMC uptake in Zimbabwe’, which indicated that when the number of VMMC service providers is adequate, VMMC uptake improves as every man has access to the service.

**Sub-theme 2: Strengthening the current programmes to motivate males to seek medical attention:** Some participants suggested that programmes to motivate males to improve their health-seeking behaviours should be introduced:

‘Males should be motivated to improve their behaviour of seeking health attention and HIV prevention’. (P6, 40 years old, Surgeon)

‘More programmes should be launched that motivate males to be more responsible and more vigilant about their health’. (P9, 37 years old, Registered Nurse)

This study found that another factor that can facilitate the improvement of VMMC uptake is to motivate or encourage males to seek medical attention and HIV prevention through campaigns. Similarly, a study conducted by Nairenge^9^ in the Zambezi region, Namibia, recommended that the VMMC team incorporate behaviour change programmes in the extant community awareness campaigns so as to improve VMMC uptake.

**Sub-theme 3: More health education to clear up myths and misconceptions:** Some participants expressed the need for more health education to clear up myths and misconceptions:

‘There is need for massive health education about VMMC to clear up myths and conceptions’. (P8, 30 years old, Health Assistant)

‘Males should be educated to clear up misconceptions attached to VMMC’. (P12, 36 years old, Health Assistant)

This study found that more health education to clear up myths and misconceptions attached to VMMC through the radio or television would have a positive impact on VMMC uptake. This is in line with a study conducted by George et al.^3^ in KwaZulu-Natal, South Africa, on the barriers to, and facilitators of, VMMC among adolescent boys. The authors recommended that strengthening the current VMMC programmes with innovative motivational methods should be implemented to help increase the uptake of VMMC by males.

**Sub-theme 4: Circumcision of boys at a young age before they become sexually active:** One participant suggested that males should be circumcised at a young age before they are sexually active:

‘Circumcision should be done starting at a tender age before the males are exposed to the risk of getting HIV’. (P3, 32 years old, Surgeon)

It emerged from this study that male circumcision should be considered at a younger age as it had a number of preventive advantages. Similarly, a study conducted by George et al.^3^ revealed that it is more beneficial to circumcise males at a young age before they experience myths about circumcision.

**Sub-theme 5: Opening more voluntary medical male circumcision centres:** The participants suggested that more VMMC centres should be opened in the region so that everyone can have equal access to services:

‘More VMMC centres should be opened in each and every region’. (P7, 34 years old, Registered Nurse)

‘I suggest that more centres for VMMC should be opened in other parts of the region so that everyone can get the services’. (P9, 37 years old, Registered Nurse)

This study ascertained that opening more VMMC centres in the region could improve uptake as everyone would have access to it. Similarly, a study conducted by Bailey^39^ in Kenya noticed that more VMMC centres would help to ensure that everyone could get the service they want, thereby improving uptake. Atkins et al.^40^ reported that one of the interventions that increased adult men’s uptake included mobile services (compared with static facilities), home-based testing with active referral follow-up, and facility-based HIV testing with enhanced comprehensive sexual education.

## Limitations and areas for further research

This study provides a broader understanding of, and insight into, the provision of VMMC services in the Kavango region. The explorative design used allowed the respondents to ‘freely narrate and interpret their experiences’, as well as suggest possible improvements in the uptake of VMMC. The use of purposive sampling and maximum variation according to age and gender, in addition to the use of a variety of professionals allocated to VMMC, assisted in obtaining diverse views. This study is limited in the sense that it focused on health workers who were used as proxy for patients. Consequently, the perspectives of patients regarding the facilitators and barriers males face in accessing VMMC were interpreted through the experiences of doctors and nurses providing VMMC. Therefore, researchers strongly recommend that further research should be conducted on exploring facilitators and barriers to the uptake of VMMC from the perspectives of patients. The study was conducted in a single setting, that is, Kavango region in the north-east part of the country. Consequently, the findings cannot be transferred to other regions of the country as the VMMC programmes in other regions are funded by different funders.

## Conclusion

The purpose of this study was to explore and describe the facilitators of, and barriers to, medical male circumcision for HIV prevention in Kavango region, Namibia. The study revealed that a number of barriers must be overcome before implementing VMMC, and before the desired number of men take advantage of VMMC. Multiple factors act as constraints to VMMC, including fear, myths and misconceptions, men’s belief that they are not at risk of getting HIV, small VMMC teams and the long distance between clients’ homes and VMMC services. This study recognises that there is a need for stakeholders in the health sector and other line ministries to collaborate in order to overcome these barriers. The study’s findings can be used to develop targeted interventions and strategies that can be used by VMMC providers to address the identified barriers.

## References

[1] WHO, UNAIDS. Voluntary medical male circumcision for HIV prevention. Geneva: WHO Publishers; 2018.

[2] Justman J, Goldberg A, Reed J, Bock N, Njeuhmeli E, Thomas AG. Adult male circumcision: Reflections on successes and challenges. J Acquir Immune Defic Syndr. 2013;63(Suppl. 2):S140–S143. 10.1097/QAI.0b013e31829875cc 23764626

[3] George G, Strauss M, Chirawu P, et al. Barriers and facilitators to the uptake of voluntary medical male circumcision (VMMC) among adolescent boys in KwaZulu–Natal, South Africa. Afr J AIDS Res. 2014;13(2):179–187. 10.2989/16085906.2014.943253 25174635

[4] WHO, UNAIDS. A frameword for voluntary medical male circumcision [homepage on the Internet]. Geneva; 2021. [cited 2023 Mar 13]. Available from: https://www.who.int/publications/i/item/WHO-HIV-2016.17

[5] Mndzebel SL, Tegegn GA. Knowledge, attitude and acceptance of voluntary male medical circumcision among male students attending Botswana University. J Public Health Epidemiol. 2015;7(1):6–14. 10.5897/JPHE2014.0671

[6] Stegman P, Stirling B, Corner B, et al. Voluntary medical male circumcision to prevent HIV: Modelling age prioritization in Namibia. AIDS Behav. 2019;23(2): 195–205. 10.1007/s10461-019-02556-y 31214866 PMC6773676

[7] WHO. WHO progress brief – Voluntary medical male circumcision for HIV prevention in priority countries of east and southern Africa. Geneva: WHO Publishers; 2018.

[8] Ministry of Health and Social Services. Namibia 2014/15 health accounts: Statistical report. Windhoek: MoHSS; 2017.

[9] Nairenge R. Assessment of knowledge, attitudes, practices and responsiveness to medical male circumcision among males in Zambezi region, Namibia [Doctoral dissertation]. Windhoek: University of Namibia; 2020.

[10] PEPFAR. Namibia country operational plan (COP) 2020 strategic direction summary, 10 March 2020 [homepage on the Internet]. [cited 2021 Sept 14]. Available from: https://na.usembassy.gov/wp-content/uploads/sites/132/COP20-Namibia-SDS-V11-FINAL.pdf

[11] Wouabe ED, Brown A. Scoping report on interventions for increasing the demand for voluntary medical male circumcision. Int Initiat Impact Eval. 2013:15–17.

[12] Hatzold K, Mavhu W, Jasi P, et al. Barriers and motivators to voluntary medical male circumcision uptake among different age groups of men in Zimbabwe: Results from a mixed methods study. PLoS One. 2014;9(5):e85051. 10.1371/journal.pone.0085051 24802746 PMC4011705

[13] World Health Organization. Male circumcision for HIV prevention – Implementing the 2017–2021 framework for voluntary medical male circumcision 27 February – 1 March 2017: Meeting report. Brazzaville: World Health Organization; 2017.

[14] Corey L, Gray GE. Preventing acquisition of HIV is the only path to an AIDS-free generation. Proc Natl Acad Sci. 2017;114(15):3798–3800. 10.1073/pnas.1703236114 28360202 PMC5393193

[15] Kibira SP, Daniel M, Atuyambe LM, Makumbi FE, Sandøy IF. Exploring drivers for safe male circumcision: Experiences with health education and understanding of partial HIV protection among newly circumcised men in Wakiso, Uganda. PLoS One. 2017;12(3):e0175228. 10.1371/journal.pone.0175228 28362880 PMC5376336

[16] Hennink M, Hutter I, Bailey A. Qualitative research methods. London: Sage; 2020.

[17] Flick U, editor. Designing qualitative research. London: Sage; 2018.

[18] Cardano M. Defending qualitative research: Design, analysis, and textualization. Abingdon, Oxfordshire: Routledge; 2020.

[19] Maree K. First steps in research. 2nd ed. Pretoria: Van Schaik; 2016.

[20] Mohajan HK. Qualitative research methodology in social sciences and related subjects. J Econ Dev Environ People. 2018;7(1):23–48. 10.26458/jedep.v7i1.571

[21] Braun V, Clarke V, Hayfield N, Terry G. Thematic analysis. In: Liamputtong P, editor. Handbook of research methods in health social sciences. Singapore: Springer; 2019. pp. 843–860.

[22] Lincoln YS, Guba EG. Naturalistic inquiry. Beverly Hills, CA: Sage; 1985.

[23] Polit DF, Beck CT. Nursing research: Generating and assessing evidence for nursing practice. Philadelphia: Lippincott Williams & Wilkins; 2017.

[24] Nguyen T. The effectiveness of online learning: Beyond no significant difference and future horizons. MERLOT J Online Learn Teach. 2015;11(2):309–319.

[25] Bell J, Waters S. eBook: Doing your research project: A guide for first-time researchers. London: McGraw-Hill Education (UK); 2018.

[26] Morris BJ, Hankins CA. Effect of male circumcision on risk of sexually transmitted infections and cervical cancer in women. Lancet Global Health. 2017;5(11): e1054–e1055. 10.1016/S2214-109X(17)30386-8 29025620

[27] Avert. Voluntary medical male circumcision for HIV prevention [homepage on the Internet]. 2016 [cited 2022 Nov 15]. Available from: http://www.avert.org/professionals/hiv-programmeming/prevention/voluntary-medical-male-circumcision

[28] Maibvise C, Mavundla TR. Reasons for the low uptake of adult male circumcision for the prevention of HIV transmission in Swaziland. Afr J AIDS Res. 2014;13(3): 281–289. 10.2989/16085906.2014.952652 25388982

[29] Maibvise C, Mavundla TR. Swazi men’s perception of the protective effect of male circumcision and its implications for HIV prevention strategy. Tanzan J Health Res. 2015;17(2):1–10.

[30] Ashengo TA, Hatzold K, Mahler H, et al. Voluntary medical male circumcision (VMMC) in Tanzania and Zimbabwe: Service delivery intensity and modality and their influence on the age of clients. PLoS One. 2014;9(5):e83642. 10.1371/journal.pone.0083642 24801882 PMC4011872

[31] Maibvise C, Mavundla TR. Medical reasons for performing adult male circumcisions in Swaziland. Afr J Nurs Midwifery. 2013;15(1):139–148.

[32] Mavhu W, Hatzold K, Ncube G, et al. Unpacking early infant male circumcision decision-making using qualitative findings from Zimbabwe. BMC Int Health Hum Rights. 2017;17(1):1–7. 10.1186/s12914-016-0111-1 28069002 PMC5223435

[33] Davis SM, Hines JZ, Habel M, et al. Progress in voluntary medical male circumcision for HIV prevention supported by the US President’s Emergency Plan for AIDS Relief through 2017: Longitudinal and recent cross-sectional programme data. BMJ Open. 2018;8(8):e021835. 10.1136/bmjopen-2018-021835 PMC6120649 30173159

[34] Masese R, Mwalabu G, Petrucka P, Mapulanga P. Key challenges to voluntary medical male circumcision uptake in traditionally circumcising settings of Machinga district in Malawi. BMC Public Health. 2021;21(1):1. 10.1186/s12889-021-11979-z 34711179 PMC8555288

[35] Curran K, Njeuhmeli E, Mirelman A, et al. Voluntary medical male circumcision: Strategies for meeting the human resource needs of scale-up in southern and eastern Africa. PLoS Med. 2011;8(11):e1001129. 10.1371/journal.pmed.1001129 22140364 PMC3226463

[36] Mahler HR, Kileo B, Curran K, et al. Voluntary medical male circumcision: Matching demand and supply with quality and efficiency in a high-volume campaign in Iringa Region, Tanzania. PLoS Med. 2011;8(11):e1001131. 10.1371/journal.pmed.1001131 22140366 PMC3226544

[37] Nyoni N, Ellion S. Assessment of knowledge, attitudes and practices of men towards voluntary male medical circumcision: Study of men between 15 to 49 years at Rundu state hospital. SCIREA J Clin Med. 2017;2(3):32–47.

[38] Mavhu W, Neuman M, Hatzold K, et al. Innovative demand creation strategies to increase voluntary medical male circumcision uptake: A pragmatic randomised controlled trial in Zimbabwe. BMJ Global Health. 2021;6(Suppl. 4):e006141. 10.1136/bmjgh-2021-006141 PMC8287600 34275877

[39] Bailey RC, Moses S, Parker CB, et al. Male circumcision for HIV prevention in young men in Kisumu, Kenya: A randomised controlled trial. Lancet. 2007;369(9562): 643–656. 10.1016/S0140-6736(07)60312-2 17321310

[40] Atkins K, Yeh PT, Kennedy CE, et al. Service delivery interventions to increase uptake of voluntary medical male circumcision for HIV prevention: A systematic review. PLoS One. 2020;15(1):e0227755. 10.1371/journal.pone.0227755 31929587 PMC6957297

